# Ceftaroline pharmacokinetics/pharmacodynamics in the hollow-fibre model of *Mycobacterium abscessus* lung disease

**DOI:** 10.5588/ijtldopen.25.0173

**Published:** 2025-09-10

**Authors:** B.E. Ferro, S. Srivastava, T. Gumbo

**Affiliations:** ^1^ Facultad de Ciencias de la Salud, Universidad Icesi, Cali, Colombia;; ^2^ Division of Infectious Diseases, Department of Medicine, School of Medicine, University of Texas at Tyler, Tyler, TX, USA;; ^3^ Department of Cellular and Molecular Biology, University of Texas Health Science Centre at Tyler, Tyler, TX, USA;; ^4^ Hollow Fiber System and Experimental Therapeutics Laboratories, Wet Lab Systems, NASOS Biotech, Dallas, TX, USA;; ^5^ IMPI Group, Harare, Zimbabwe.

**Keywords:** avibactam, Bla_Mab_, cefoxitin, anti-microbial resistance, combination therapy

## Abstract

**BACKGROUND:**

Guideline-based therapy (GBT) of *Mycobacterium abscessus* (MAB) lung disease (LD) achieves a sputum culture conversion rate in ∼35% of patients. The β-lactam antibiotics, imipenem and cefoxitin, are the cornerstone of GBT. However, a 1-h half-life means multiple infusions per day, which is difficult for months-long therapy.

**METHODS:**

As an alternative, we tested ceftaroline–avibactam, with a 3-h half-life, in the hollow-fibre model system of MAB-LD (HFS-MAB). Eight ceftaroline–avibactam exposures were administered twice daily based on human intrapulmonary pharmacokinetics. Next, we compared GBT (cefoxitin–clarithromycin–amikacin) to standard-dose and to high-dose ceftaroline–avibactam–tigecycline–moxifloxacin regimens, in the HFS-MAB, mimicking the intrapulmonary pharmacokinetics of all drugs.

**RESULTS:**

Ceftaroline–avibactam maximal microbial kill was 0.72 log_10_ colony-forming units (CFUs) per millilitre below day 0 burden (stasis). The ceftaroline pharmacokinetic–pharmacodynamic parameter linked to microbial kill and anti-microbial resistance was 0–24-h area under the concentration–time curve (AUC_0–24_) to minimum inhibitory concentration. GBT killed 1.21 ± 0.25 log_10_ CFU/mL versus 0.45 ± 0.42 log_10_ CFU/mL below stasis for high-dose ceftaroline combination (*P* = 0.20). In combination regimens, proportion of the ceftaroline-resistant subpopulation was 41.22 ± 13.11 times lower than the cefoxitin-resistant subpopulation (*P* = 0.002).

**CONCLUSION:**

Ceftaroline–avibactam administered twice a day would be an adequate replacement for the four-times-a-day cefoxitin treatments for MAB-LD.

Guideline-based therapy (GBT) for *Mycobacterium abscessus* (MAB) lung disease (LD) consists of treatment with at least three active drugs, including one to two from the injectable group of β-lactams (imipenem or cefoxitin), amikacin, and tigecycline, and two from either macrolides, clofazimine, linezolid, or inhaled amikacin.^1^ However, the two β-lactams have a half-life of an hour or less, necessitating multiple infusions per day, which is difficult for months-long therapy.^1–3^ In addition to not being patient-friendly, the GBT achieves a sputum culture conversion rate (SCC) in only 20%–35% of patients at the expense of adverse events in about 79% of the patients.^4,5^

There are concerted efforts to find which of the drugs in the combination have any effect at all (i.e., have biologic activity) and to find new drugs that could replace those that do not. We have focused on the β-lactams and tetracyclines using pharmacokinetic (PK)–pharmacodynamic (PD) approaches that employ drug exposure indices such as % time concentration that persists above minimum inhibitory concentration (MIC) (%T_MIC_), peak concentration (C_max_) to MIC, and 0–24-h area under the concentration–time curve (AUC_0–24_) to MIC, which allows translation to clinical doses.^6–9^ Here, we investigated the potential role of the fifth-generation cephalosporin, ceftaroline, in the hollow-fibre system model of MAB-LD (HFS-MAB).^10^

The HFS-MAB can mirror the microbial response rates seen in patients.^3,6,7,9,11,12^ In the HFS-MAB, amikacin monotherapy killed close to 0 CFU/mL, GBT (clarithromycin–cefoxitin–amikacin) killed only 1.2 log_10_ CFU/mL, and tigecycline killed 1.36 log_10_ CFU/mL, below day 0 bacterial burden (stasis) or *B*_0_.^6,9,11,12^ Omadacycline killed 2.09 log_10_ CFU/mL below stasis in the HFS-MAB, at exposures that could be achieved by 300 mg/day.^7^ Patient/population, intervention, comparison, and outcome (PICO) analyses demonstrated 80% SCC for omadacycline 300 mg/day–based combinations, and in a recent news release of a randomised controlled study, omadacycline 300 mg/day as monotherapy achieved an SCC of 56%.^7,13^ We have also used the same HFS-MAB plus PICO approach to investigate another β-lactam, imipenem.^3^

β-Lactams inhibit MAB cell wall synthesis by binding to D,D-transpeptidases and L,D-transpeptidases 1 to 5 (Ldt_Mab1_ to Ldt_Mab5_).^14^ However, all MAB subspecies possess the β-lactamase, Bla_Mab_.^15,16^ The balance between β-lactam binding affinity to the penicillin-binding proteins versus the sum of hydrolysis by bacterial β-lactamase and patient drug clearance (and thus, half-life) accounts for efficacy. This balance could be tipped by two approaches: first, the use of β-lactams with longer half-lives such as ceftaroline, and second, the use of β-lactamase inhibitors (BLIs) such as avibactam. Avibactam rapidly forms covalent bonds with Bla_Mab_, D,D-transpeptidase, Ldt_Mab1_, Ldt_Mab2_, and Ldt_Mab4_, while ceftaroline binds the same targets except Bla_Mab_ for which it is a poor substrate.^14^ It is unclear if adding avibactam to ceftaroline would be beneficial for MAB-LD therapy given that ceftaroline is a poor substrate of Bla_Mab_.^14^ Ceftaroline fosamil is a prodrug that is rapidly converted to the active moiety by plasma phosphatases.^17^ Ceftaroline PKs include a half-life of 2.7–3 h in plasma and epithelial lining fluid (ELF), and an ELF/plasma penetration ratio of 24%.^18,19^ Here, we tested ceftaroline plus avibactam in the HFS-MAB.

## METHODS

### Materials and bacterial isolates

Drugs were purchased from Baylor University Medical Center Pharmacy (Dallas, TX, USA). All other reagents were purchased from Sigma-Aldrich (St. Louis, MO, USA). Antibiotics were either stock prepared or dissolved, sterile filtered, and diluted to the desired concentrations in broth. MAB American Type Culture Collection (ATCC) 19977 cultures were grown to logarithmic phase in Middlebrook 7H9 broth supplemented with 10% oleic acid–albumin–dextrose–catalase for each experiment. Cultures were incubated at 30°C and sub-cultured every 72 h to maintain the logarithmic-phase growth.

### MIC and mutation frequency

Broth microdilution MICs were performed using cation-adjusted Mueller Hinton Broth (CAMHB).^20,21^ Mutation frequency was determined for the inoculum by spreading 0.2 mL on each of 20 Middlebrook agar plates supplemented with 3× ceftaroline MIC concentration. All cultures were incubated for 72 h at 30°C before MICs were recorded or samples were processed for CFU estimation.

### Ceftaroline activity in 24-well plates

We performed a 24-well study of ceftaroline with drug (concentrations: 0, 4, 8, 16, 32, 64, 128, and 256 mg/L) diluted in CAMHB, which was then co-incubated 50/50 (V/V) in 24-well plates, with and without 75 IU/L of human alkaline phosphatase (ALP). A second study examined combinations of 22 mg/L of ceftaroline, 88 mg/L of ceftazidime, and 4 mg/L of avibactam. Cultures were incubated for 72 h at 30°C followed by processing for CFU estimation.

### Exposure-response HFS-MAB study

We used the previously developed HFS-MAB, using the cellulosic hollow-fibre cartridges from FiberCell Systems (Cat#C8008; Frederick, MD, USA).^6–9,11,12^ We examined eight different ceftaroline target exposures to achieve 0%, 10%, 20%, 40%, 60%, 80%, and 100%-time above MIC (%T_MIC_), infused over an hour, every 12 h (Q12), with a half-life of 3 h, over 28 days of study. The inflow rate was 0.4 mL/min. We sampled the central compartment of the each HFS-MAB unit over 48 h for validation of the PK profile. Our studies demonstrated that ceftaroline equilibrates between the central and peripheral compartment in less than 5 min. The peripheral compartments were sampled on days 0, 1, 2, 3, 5, 7, 10, 14, 21, and 28 for enumeration of the total bacterial burden as well as ceftaroline-resistant subpopulation. To capture ceftaroline-resistant subpopulation, Middlebrook 7H10 agar was supplemented with 3× ceftaroline MIC (48 mg/L) in combination with 4 mg/L for avibactam, with and without the efflux pump inhibitor, reserpine. The 3× MIC threshold was chosen to capture the low-level drug-resistant subpopulation that initiates the anti-microbial resistance (AMR) arrow of time process, before gene mutations appear.^22^

### Combination therapy studies in the HFS-MAB

We tested the efficacy of GBT versus ceftaroline–avibactam combination regimens in the HFS-MAB. All drugs were infused over 1 h, except amikacin which was infused over 30 min. We compared two ceftaroline-based regimens (human equivalent standard dose [SD] 600 mg twice a day at AUC_0–24_/MIC = 10 [%T_MIC_ = 25]) vs. high dose (HD) of 1,800 mg Q8 at AUC_0–24_/MIC = 30 (%T_MIC_ = 75) combined with tigecycline (50 mg/day SD and 500 mg/day HD) plus moxifloxacin (human equivalent SD of 400 mg/day or HD of 600 mg/day). The GBT was administered with the following intrapulmonary PK: clarithromycin (AUC_0–24_/MIC = 5.3), amikacin (C_max_/MIC = 4), and cefoxitin (%T_MIC_ = 100%). The dose of avibactam used was equivalent to 1,000 mg/day to achieve a C_max_ of 50 mg/L, administered twice a day simultaneously with ceftaroline. Cefoxitin was administered four times a day while clarithromycin and amikacin were administered once a day. The central compartment was sampled 12 times over 48 h to measure concentrations of each drug using published assays.^3,6–9,11,12^ Total and AMR bacterial burden in the peripheral compartment were cultured as in the exposure–effect study above.

### PK/PD analyses

Drug exposure versus effect data were analysed using the inhibitory sigmoid E_max_ model for the total MAB population as well as the quadratic function of Gumbo et al. for AMR populations.^3,6,7,9,11,12^ The PK/PD parameter linked to effect in these two models was chosen using corrected Akaike information criteria (cAIC).^23^

## RESULTS

### MICs and ceftaroline activity in 24-well plates with and without avibactam

**Figure 1.**
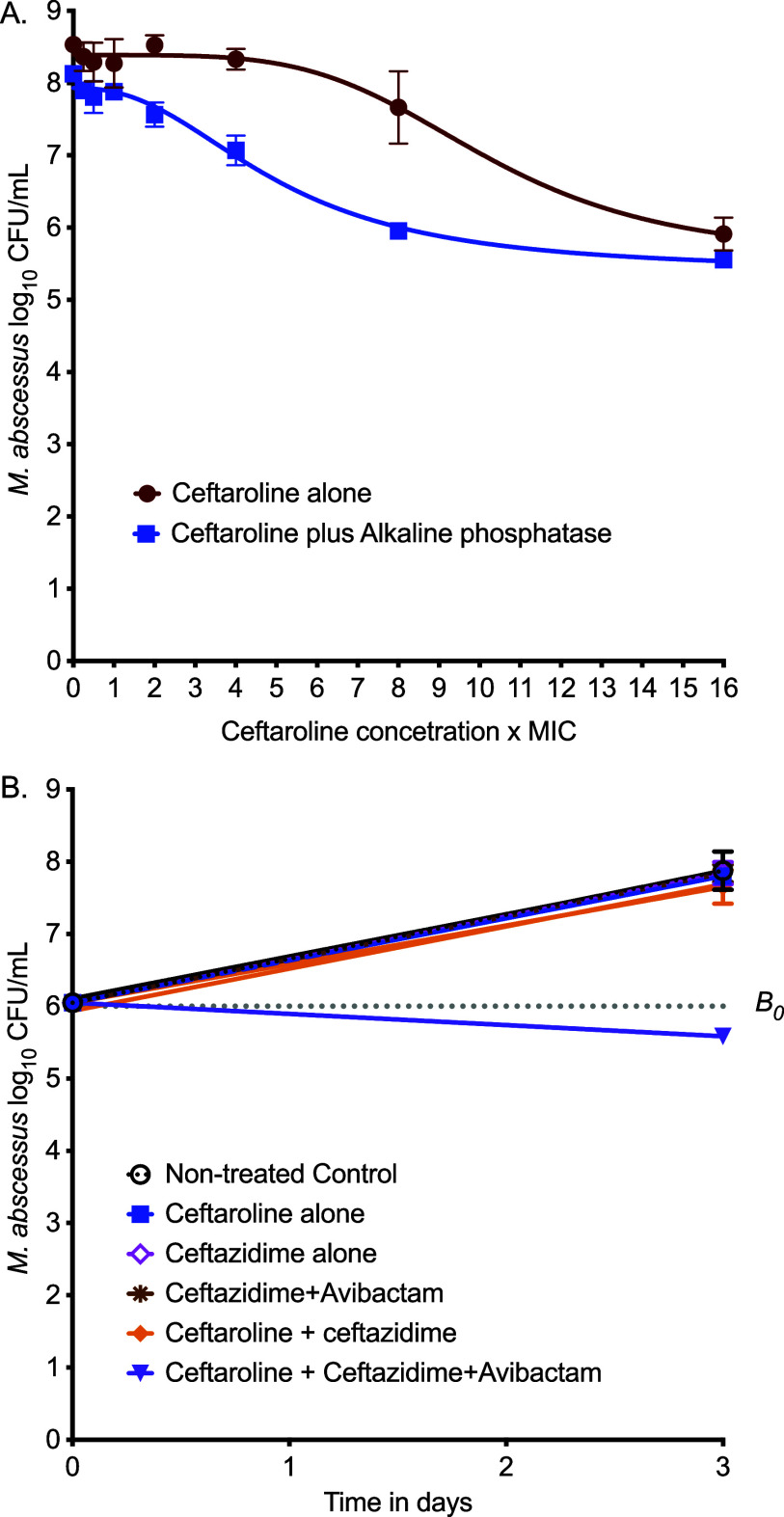
Ceftaroline, avibactam, and dual β-lactam static concentration versus effect studies. **A:** Ceftaroline concentration is given as a multiplicity of MIC and is unitless (mg/L divided by mg/L). **B:** *B*_0_ is bacterial burden on day 0 or stasis. MIC = minimum inhibitory concentration.

The MICs for the different drugs against MAB were as shown in Supplementary Data Table S1. Figure 1A shows the inhibitory sigmoid E_max_ model curves in the first 24-well study. The E_max_ for ceftaroline alone was 2.59 log_10_ CFU/mL versus 2.57 log_10_ CFU/mL with ALP. However, the concentration mediating 50% of E_max_ (EC_50_) was 148.6 mg/L (95% confidence intervals [95% CI]: 125.8–171.5) for ceftaroline versus 76.79 mg/L (95% CI: 67.14–86.44) for ceftaroline fosamil plus ALP, demonstrating successful conversion from prodrug to active moiety. Thus, ceftaroline fosamil with 75 IU/L ALP was used in subsequent experiments. The pattern of kill, whereby E_max_ was not achieved before 16 times the MIC, strongly suggests that the ceftaroline effect was concentration-driven instead of time-driven.^24^ Figure 1B shows the results of ceftaroline alone versus ceftazidime–avibactam versus combinations. All drugs had no effect, either alone or as dual combinations. However, the double β-lactams combination of ceftaroline–ceftazidime–avibactam killed MAB below stasis.

### Ceftaroline–avibactam PK/PD studies in the HFS-MAB

**Figure 2.**
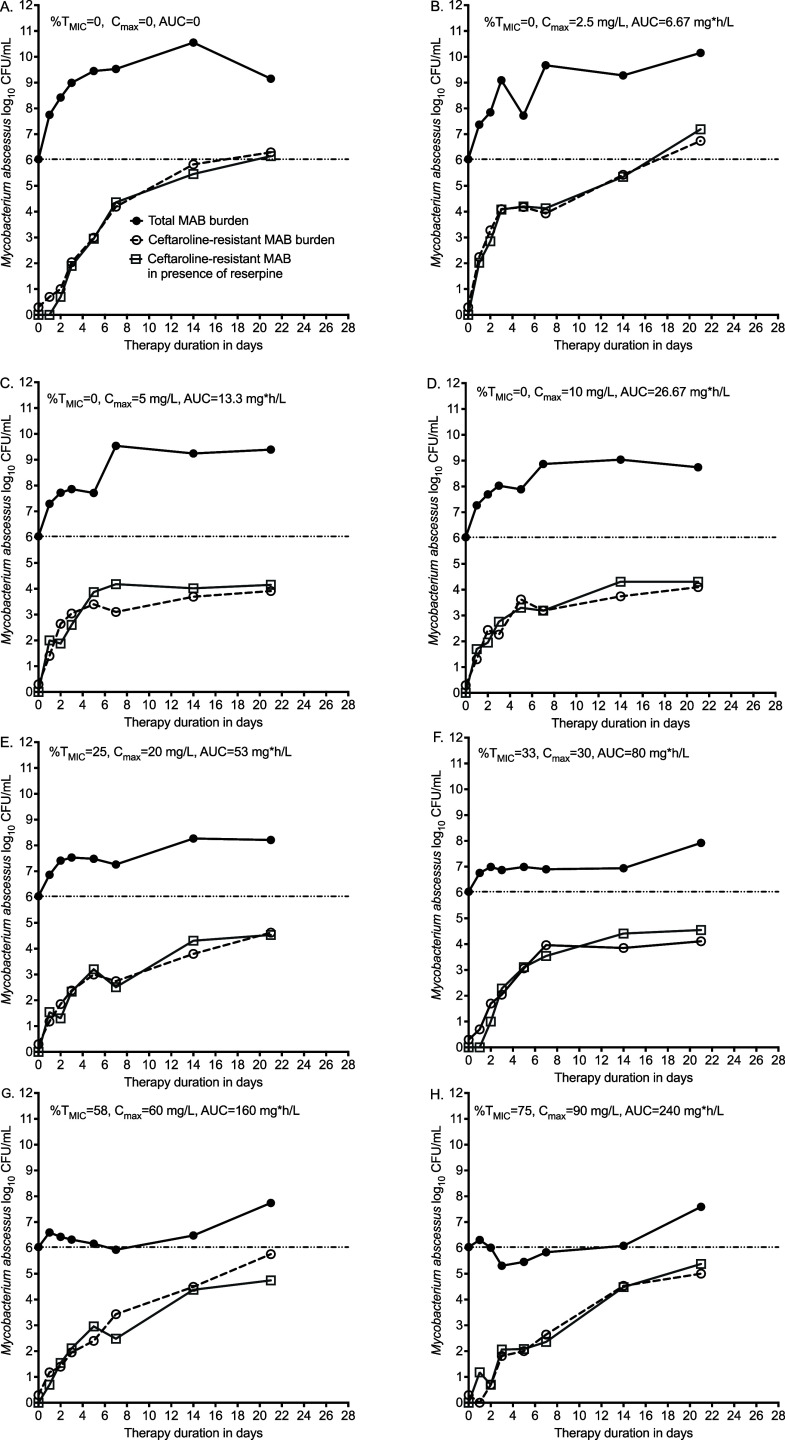
Ceftaroline microbial kill and anti-microbial resistance in the HFS-MAB. Time-kill and time-resistance curves. Symbols are observed colony-forming units, and line graphs are simple point to point. **A:** The shapes of the non-treated total MAB burden and drug-resistant colony-forming units (CFUs) with time are parallel and reflect the baseline mutation frequency. **B–F:** As exposures increased, both total bacterial burden and drug-resistant subpopulation were lower than non-treated controls. **G and H:** At exposures such as %T_MIC_ = 58 and AUC_0–24_/MIC = 10, the biphasic nature of response became more pronounced. AUC = under the concentration; HFS = hollow-fibre model system; MAB = *Mycobacterium abscessus*; MIC = minimum inhibitory concentration*.*

The ceftaroline–avibactam mutation frequency for the inoculum was 8.83 ± 2.365 × 10^−7^. The target exposures were achieved. The time-kill curves for each dose, for both the total bacterial population and ceftaroline-resistant burden, are shown in Figure 2A–H. There was a concentration-dependent decrease in total bacterial burden in a biphasic manner. The highest exposures (Figure 2H) of ceftaroline killed MAB 0.72 log_10_ CFU/mL below stasis. However, the microbial kill was terminated by AMR in all exposures. In the non-treated controls, the AMR subpopulations paralleled the total population, reflecting the mutation frequency. In the ceftaroline–avibactam treated systems, the size of the AMR bacterial burden decreased as the concentrations increased, confirming that the effect was concentration-dependent. As concentrations increased, the AMR subpopulation also started to increase, shown in Figure 2F. This observation is consistent with the AMR-arrow of the time model, which hitherto was assumed to be initiated by early efflux pump induction.^22,25^ Nevertheless, Figure 2 shows that AMR population did not change in the presence of reserpine, suggesting a minimal role for some types of efflux.

**Figure 3.**
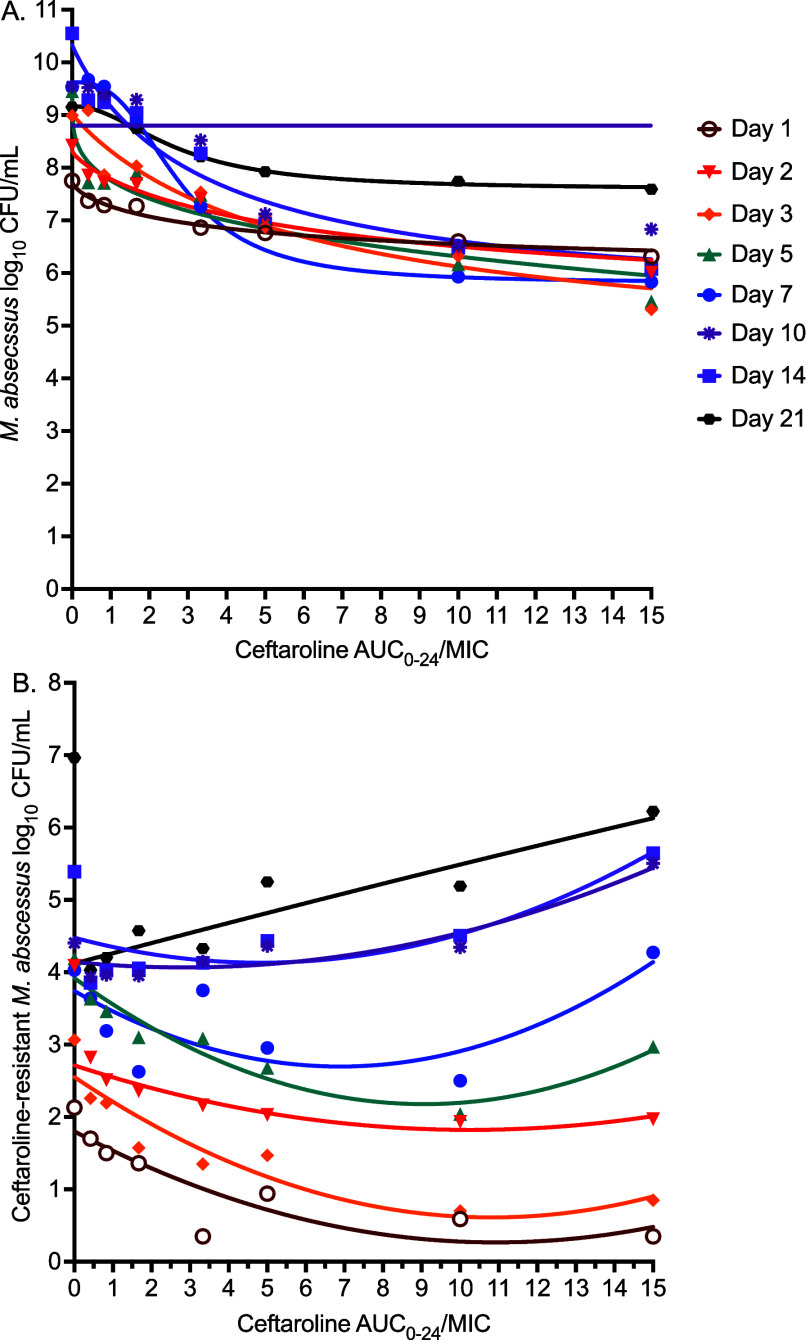
Pharmacokinetic/pharmacodynamic modelling in the HFS-MAB. Symbols are the observed colony-forming units, while line graphs are the model fit. **A:** Inhibitory sigmoid E_max_ model curves for each sampling day for AUC_0–24_/MIC ratio versus bacterial burden. **B:** Quadratic function model in the antibiotic resistance arrow of time. AUC = under the concentration; HFS = hollow-fibre model system; MAB = *Mycobacterium abscessus*; MIC = minimum inhibitory concentration*.*

In Supplementary Data Table S2, inhibitory sigmoid E_max_ modelling for microbial kill demonstrated that AUC_0–24_/MIC had the lower cAIC scores than %T_MIC_, suggesting a better PK/PD link for ceftaroline with AUC_0–24_/MIC for microbial kill. Since this was not a dose-fractionation study, the C_max_/MIC was not explored as an independent PK/PD driver because it covaries with AUC_0–24_/MIC. Figure 3A shows the inhibitory sigmoid E_max_ curves for each sampling day for AUC_0–24_/MIC, and the model parameter estimates are shown in Supplementary Data Table S3. The EC_50_s were virtually identical between sampling days as demonstrated by a % coefficient of variation of 38.2%. Therefore, we aggregated the EC_50_s. The EC_50_ mean ± standard error (SE) was AUC_0–24_/MIC of 4.55 ± 0.61 (%T_MIC_ = 39.75±7.64), while EC_80_ was AUC_0–24_/MIC of 9.84 ± 1.32 (%T_MIC_ = 66.50 ± 12.78).

In Supplementary Data Table S2, quadratic function modelling of AMR, AUC_0–24_/MIC showed lower cAIC scores compared with %T_MIC_. Thus, based on the data presented here, for % AMR subpopulation, we concluded AUC_0–24_/MIC as a better PK/PD link for the ceftaroline resistance.^26^ Figure 3B shows the quadratic function curves for resistance versus AUC_0–24_/MIC, and Supplementary Data Table S3 lists the model parameter estimates for exposures associated with AMR. The mean ± SE AUC_0–24_/MIC associated with the nadir or lowest % of the ceftaroline-resistant subpopulation (i.e., AMR suppression) for each day was calculated as 2.569 ± 0.651 (%T_MIC_ = 18.76 ± 2.94).

### Ceftaroline–avibactam-based combination therapy in the HFS-MAB

**Figure 4.**
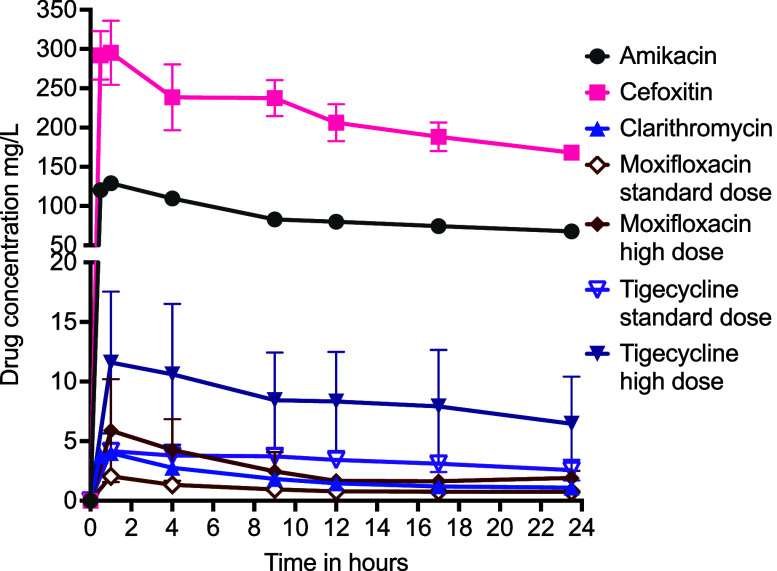
Concentration–time profiles of drugs achieved in combination therapy. Concentrations of different drugs in combination therapy measured in the hollow fibre after the last dose of the experiment. Shown are mean concentrations while error bars are standard deviation. Different drugs achieved different half-lives and peak concentrations, similar to those seen in patients.

**Figure 5.**
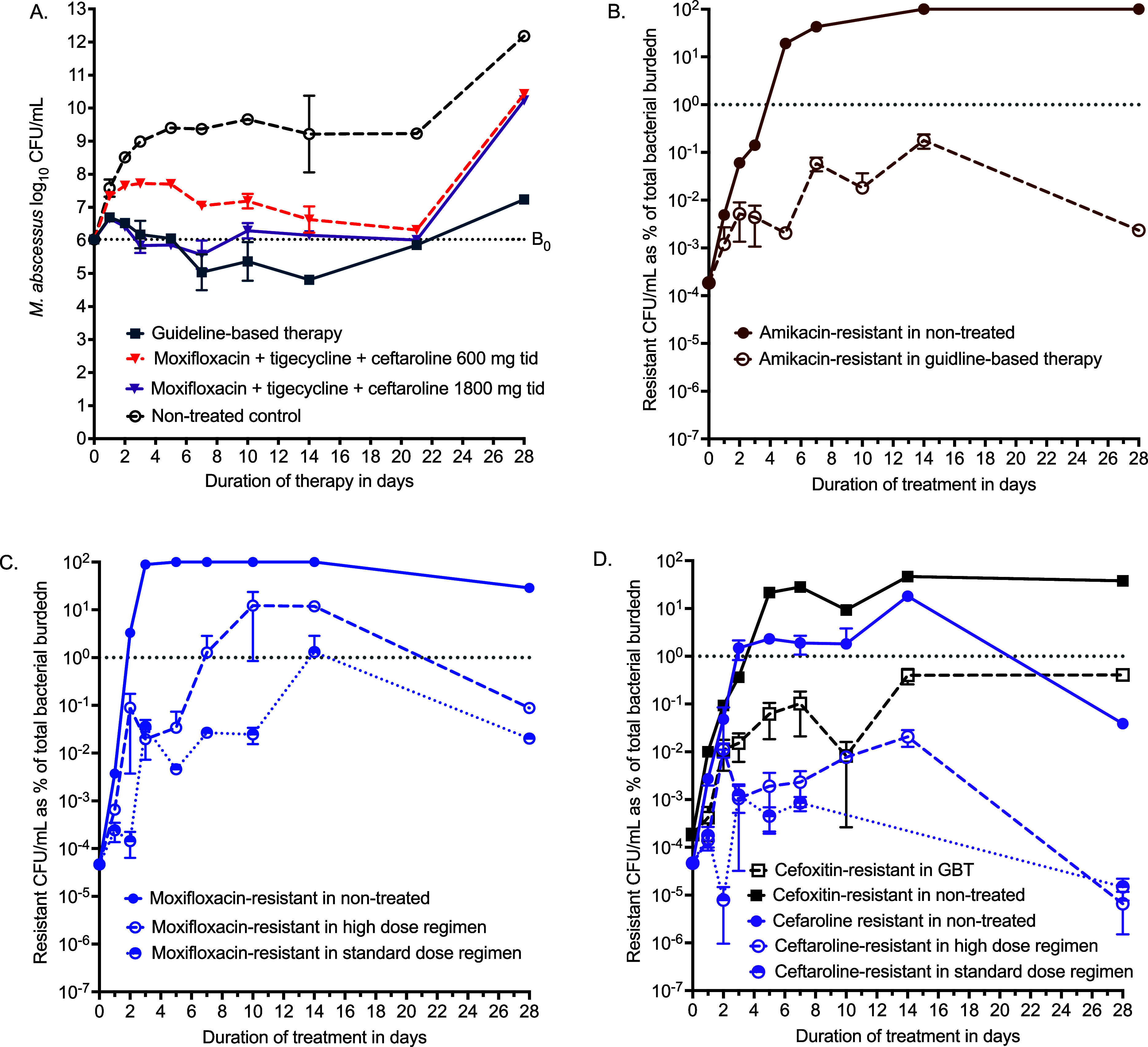
Combination therapy microbial kill and anti-microbial resistance. Symbols are mean CFU/mL while error bars are standard deviations. **A:** Total bacterial burden (log_10_ CFU/mL) shows that guideline-based therapy (GBT) performed the best. **B–D:** Drug-resistant CFU/mL divided by total burden CFU/mL, expressed as %. **B:** In guideline-based therapy-treated systems, clarithromycin and tigecycline reduced amikacin-resistance %. **C:** Moxifloxacin resistance was lower in the ceftaroline and tigecycline combination. **D:** Cephalosporin resistance. CFU = colony-forming unit; tid = three times‐a‐day.

The measured drug concentrations in the HFS-MAB are shown in Figure 4, and the resulting PK/PD exposures are summarised in Supplementary Data Table S4. Microbiological kill curves with different combinations are shown in Figure 5A. The clarithromycin–amikacin–cefoxitin combination killed 1.21 ± 0.25 log_10_ CFU/mL below stasis compared with 0.45 ± 0.42 log_10_ CFU/mL with the high-dose ceftaroline combination regimen (*P* = 0.200), but the effect was transient and was above stasis by end of the experiment. The standard-dose ceftaroline combination regimen failed to kill below stasis at any point. Figure 5B–D shows that microbial kill for all regimens was upended by AMR, expressed as percentage of drug-resistant population to total CFU/mL on each sampling day. Figure 5B and 5C shows that the combination regimen protects against resistance to companion drugs for amikacin and moxifloxacin. Figure 5D shows the cephalosporin-resistant subpopulations in the different regimens. First, when cefoxitin-resistant percentage in GBT was compared to ceftaroline-resistant percentage in the moxifloxacin–tigecycline–ceftaroline regimens on each sampling day using the mixed-effects model, the ceftaroline-resistant subpopulation was 41.22 ± 13.11 times lower than cefoxitin (*P* = 0.002), and this ratio widened with duration of therapy (*P* = 0.007). This suggests a lower propensity for ceftaroline–avibactam to develop AMR compared with cefoxitin. Second, Figure 5D shows that treatment with tigecycline and moxifloxacin reduced the ceftaroline-resistant proportion compared with non-treated, which means the companion drugs reduced ceftaroline resistance.

## DISCUSSION

First, GBT recommends either cefoxitin or imipenem as part of the initial treatment of MAB-LD.^1^ In one retrospective study of 244 patients, cefoxitin and meropenem were shown to not contribute to SCC, while imipenem contributed.^5^ We have shown that imipenem/relebactam is one of the most effective β-lactam/BLI antibiotics, demonstrating biologic activity in the HFS-MAB and in patients.^3^ Here, we demonstrated ceftaroline–avibactam biologic activity in the HFS-MAB, with a microbial kill better than amikacin’s 0 CFU/mL in the HFS-MAB.^11^ Moreover, the ceftaroline-resistant MAB subpopulation was multiple-fold lower than for cefoxitin. Furthermore, ceftaroline–avibactam twice daily dosing will be more convenient than cefoxitin 6-hourly dosing schedule. Therefore, we propose replacing four-times-a-day cefoxitin with twice-a-day ceftaroline–avibactam.^5^

Second, the PK/PD parameter linked to the ceftaroline–avibactam effect did not ‘wobble’ during the 28 days of the study, as has been encountered in slow-growing mycobacteria in the HFS and in patients.^27–30^ Thus, EC_50_ and EC_80_ targets were robust and precise. We also found that the ceftaroline efficacy was AUC_0–24_/MIC linked instead of %T_MIC_, as is often the case with β-lactams.^24^ Indeed, AMR was also linked to AUC_0–24_/MIC. The reasons are unclear but could include the influence of avibactam on the PK/PD parameter linked to microbial kill and AMR. Regardless, this AUC_0–24_/MIC-dependent efficacy likely explains why a twice-a-day dosing schedule of ceftaroline was able to kill MAB in the HFS-MAB below stasis, even with a 3-h half-life.

Finally, we studied ceftaroline–avibactam as a component of combination, to identify important principles for use in future novel regimens. This work was performed before our omadacycline work for MAB-LD, at a time when tigecycline was the most effective antibiotic for MAB.^7^ On the other hand, moxifloxacin has since demonstrated poor effect in the HFS-MAB.^6^ The best combination may be a dual β-lactam regimen. In one study by Pandey et al. with 30 MAB isolates, ceftaroline demonstrated an MIC_50_ of 16 mg/L and MIC_90_ of 32 mg/L, avibactam reduced this by 3–4 tube dilution to MIC_50_ of 1 mg/L and MIC_90_ of 4 mg/L, while ceftaroline/avibactam–ceftazidime reduced this by 5–6 tube dilution to an MIC_50_ of 0.25 mg/L and an MIC_90_ of 1 mg/L.^31^ The possible explanation for the shift in the MIC in combination of avibactam could be that avibactam alters the enzymatic activity of the MAB β-lactamase.^31,32^ Since ceftazidime–avibactam 1) has a half-life and lung penetration ratio that match those of ceftaroline making twice-a-day infusion regimen a good possibility and 2) reduces the ceftaroline MICs by 5–6 tube dilutions, we propose testing a twice-a-day ceftaroline–ceftazidime-avibactam dual β-lactam regimen, combined with twice-a-day epetraborole and once-a-day omadacycline.^14,31,33,34^

Our study has several limitations. First, drugs used in the combination therapy study such as moxifloxacin and tigecycline, which looked promising when the combination therapy HFS-MAB was performed, have now been surpassed by such drugs as omadacycline and epetraborole. Second, MIC work will need to be performed with a large number of different subspecies of MAB to enable dose-finding Monte-Carlo experiments. Third, in the past, we used to do PK sampling over 24–48 h, as we did here. However, interactions with regulatory authorities have led us to do PK sampling throughout the study. Finally, our review of data for regulatory submission shows that HFS-MAB replicates are needed even for exposure–effect studies.

Despite these limitations, we show that ceftaroline–avibactam can replace cefoxitin and should be studied in combination therapy for MAB-LD.

## Supplementary Material


