# Supplementary material for: Ceftaroline pharmacokinetics/pharmacodynamics in the hollow-fibre model of Mycobacterium abscessus lung disease
Source: IJTLD Open. 2025 Sep 10;2(9):519–26. doi: 10.5588/ijtldopen.25.0173 (PMC12435454; doi:10.5588/ijtldopen.25.0173)
Supplement: Supplementary file 1 [file ijtldopen25-0173_supplementarydata1.pdf]

Supplementary Table S1. Minimum inhibitory concentration (MIC) of the drugs used in the study.

**Antibiotic**	**MIC (mg/L)**
Amikacin	32
Cefoxitin	16
Ceftaroline	32
Ceftaroline/avibactam	16
Clarithromycin	8
Moxifloxacin	2
Tigecycline	4

Supplementary Table S2. Inhibitory sigmoid maximal effect model parameter comparison by corrected Akaike Information Criteria Scores.

	**Day 1**	**Day 2**	**Day 3**	**Day 5**	**Day 7**	**Day 14**	**Day 21**
**Microbial kill**							
$\%T_{MIC}$	-5.868	1.382	13.45	**3.969**	2.601	13.99	10.75
AUC/MIC	**-16.72**	**-4.343**	**7.492**	28.70	**-9.752**	**9.108**	**-15.10**
**Resistance**							
$\%T_{MIC}$	**-14.89**	-13.30	-33.46	-19.25	-3.477	-24.94	-10.89
AUC/MIC	-14.77	**-20.41**	**-36.27**	**-34.92**	**-10.05**	**-27.39**	**-13.09**

AUC/MIC showed the lowest AIC scores for all sampling days compared to the $\%T_{MIC}$, hence determined the PK/PD indices associated with ceftaroline efficacy against MAB.

Supplementary Table S3. Ceftaroline PK/PD parameter estimates (PE) and standard error (SE) in the HFS-MAB.

	Day 1		Day 2		Day 3		Day 5		Day 7		Day 10		Day 14		Day 21	
	PE	SE	PE	SE	PE	SE	PE	SE	PE	SE	PE	SE	PE	SE	PE	SE
**MICROBIAL KILL**																
**E_{con} log_{10} CFU/mL**	7.727	0.099	8.314	0.192	9.019	0.357	8.774	0.3765	9.622	0.042	9.439	0.076	10.330	0.404	9.166	0.033
**E_{max} log_{10} CFU/mL**	1.859	0.478	3.280	1.200	4.694	1.579	4.080	0.7336	3.805	0.063	2.770	0.134	5.052	1.162	1.582	0.065
**H**	0.721	0.229	0.804	0.314	0.965	0.433	0.940	0.360	2.802	0.197	5.693	1.235	0.969	0.368	2.050	0.236
**EC_{50} AUC/MIC**	4.644	3.511	7.713	7.171	6.079	4.371	5.234	3.593	2.787	0.071	3.759	0.161	3.460	1.770	2.709	0.153
**R^2**	0.975		0.967		0.954		0.891		0.999		0.993		0.959		0.998	
**RESISTANCE (%)**																
**% in non-treated x 10^{-3}**	0.161	0.071	0.630	0.097	1.287	0.376	9.126	4.725	6.861	3.892	78.639	42.924	376.078	249.963	61.617	107.495
**AUC/MIC at lowest %**	3.976		6.535		2.333		2.445		1.067		1.882		2.253		0.778	
**R^2**	0.130		0.835		0.999		0.998		1.00		1.00		0.999		0.995	

Supplementary Table S4. Combination therapy exposures as achieved in the HFS-MAB.

	Amikacin	Cefoxitin	Clarithromycin	Moxifloxacin 400 mg	Moxifloxacin 600 mg	Tigecycline Standard Dose	Tigecycline High Dose
C_{max}/MIC	4.03	18.45	0.50	1.03	2.56	0.93	2.52
AUC_{0-24}/MIC	63.34	310.63	5.45	13.1	36.41	20.01	51.63
$\%T_{MIC}$	100	100	0	12.5	70.83	12.5	100
